# Multiplex PCR for rapid diagnosis and differentiation of pox and pox-like diseases in dromedary Camels

**DOI:** 10.1186/s12985-015-0329-x

**Published:** 2015-07-07

**Authors:** Abdelmalik I Khalafalla, Khalid A Al-Busada, Ibrahim M El-Sabagh

**Affiliations:** Camel Research Center, King Faisal University, Al-Ahsa, 31982 Saudi Arabia; Department of Microbiology, Faculty of Veterinary Medicine, University of Khartoum, P.O. Box 32, Shambat, Sudan; Department of Biochemistry, Physiology and Pharmacology, College of Veterinary Medicine and Animal Resources, King Faisal University, Al-Ahsa, 31982 Saudi Arabia; Central Biotechnology Laboratory, Faculty of Veterinary Medicine and Animal Resources, King Faisal University, Al-Ahsa, 31982 Saudi Arabia; Department of Virology, Cairo University, Giza, 12211 Egypt

**Keywords:** Development, Multiplex PCR, Diagnosis, Pox and pox-like diseases, Camels

## Abstract

**Background:**

Pox and pox-like diseases of camels are a group of exanthematous skin conditions that have become increasingly important economically. Three distinct viruses may cause them: camelpox virus (CMLV), camel parapox virus (CPPV) and *camelus dromedary* papilloma virus (CdPV). These diseases are often difficult to differentiate based on clinical presentation in disease outbreaks. Molecular methods such as PCR targeting species-specific genes have been developed and used to identify these diseases, but not simultaneously in a single tube. Recently, multiplex PCR has gained reputation as a convenient diagnostic method with cost-and timesaving benefits.

**Methods and results:**

In the present communication, we describe the development, optimization and validation of a multiplex PCR assay able to detect simultaneously the genome of the three viruses in one single test allowing for rapid and efficient molecular diagnosis. The assay was developed based on the evaluation and combination of published and new primer sets and was validated with viral genomic DNA extracted from known virus strains (*n* = 14) and DNA extracted from homogenized clinical skin specimens (*n* = 86). The assay detects correctly the target pathogens by amplification of targeted genes, even in case of co-infection. The method showed high sensitivity, and the specificity was confirmed by PCR-product sequencing.

**Conclusion:**

This assay provide rapid, sensitive and specific method for identifying three important viruses in specimens collected from dromedary camels with varying clinical presentations.

## Background

**Fig. 1:**
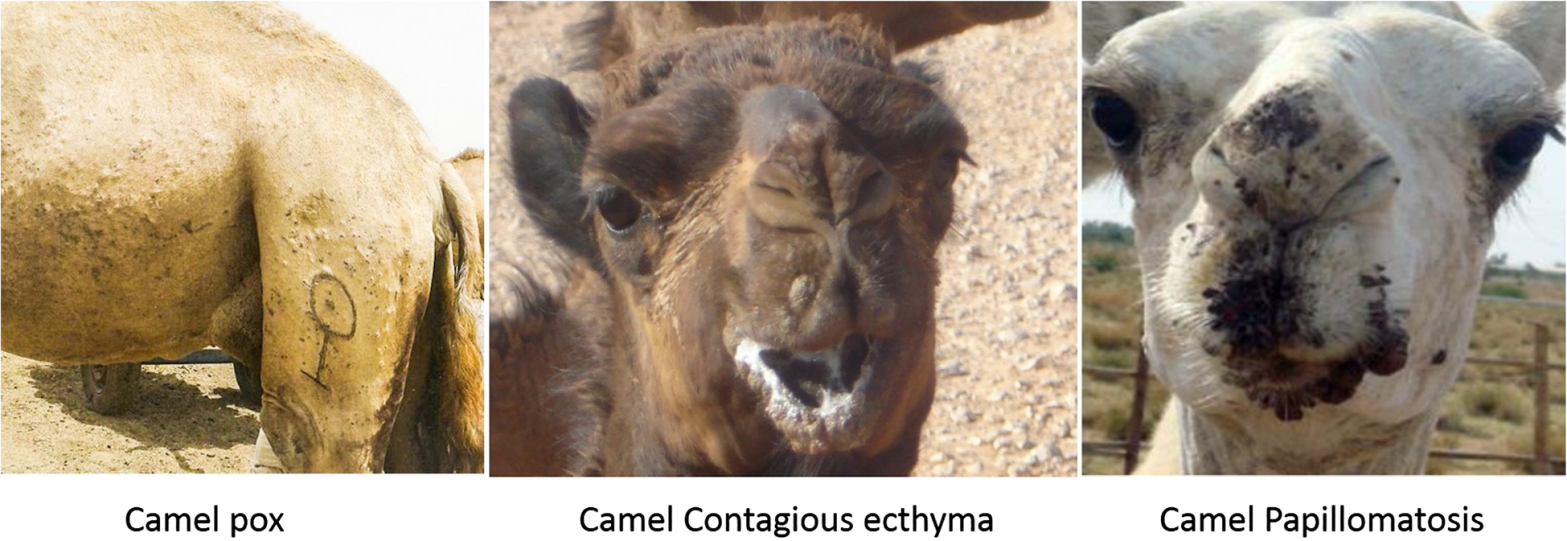
Clinical manifestation of pox- and pox-like diseases of camels

Pox and pox-like diseases of camels are a group of exanthematous skin conditions that have become increasingly important economically [1]. They include camelpox, which is caused by the *Camelpox virus* (CMLV), of the genus *Orthopoxvirus* (OPV) and camel contagious ecthyma (CCE) also named Auzdik disease or orf in camels, which is caused by a tentative member of the genus *Parapoxvirus* (PPV), both viruses belong to the subfamily *Chordopoxvirinae* and the family *Poxviridae*. The group also includes warts or camel papillomatosis caused by the *camelus dromedary* papilloma virus (CdPV) [2]. These three viruses induce in dromedary camels (*camelus dromedarius*) a proliferative cutaneous dermatitis that vary in shape, pattern and distribution of the lesion (Fig. 1).

Camel pox is a highly contagious viral skin disease and occurs in almost every country in which camel husbandry is practiced [3]. The clinical signs of camelpox vary from acute to mild infection and may include fever, lymph node enlargement, face edema, lachrymation, pendulous lips and pox lesions. Papules and vesicles first appear on the lips and nostrils and later involve the whole head, neck, buttock, abdomen, legs and groin [4]. Outbreaks have been reported in Asia (Bahrain, Iran, Iraq, Oman, Saudi Arabia, United Arab Emirates, Yemen, Syria, Afghanistan, southern parts of Russia and India, and Pakistan) and in Africa (Algeria, Egypt, Ethiopia, Kenya, Mauritania, Morocco, Niger, Somalia and Sudan) [5, 6]. The disease is endemic in these countries and a pattern of sporadic outbreaks occurs with a rise in seasonal incidence usually during the rainy season [5]. The disease was reported in the past five years from Saudi Arabia [7], India [3, 8], Ethiopia [9] and Iran [10].

Localized lesions, mainly around the mouth and nares, characterize camel contagious ecthyma (CCE) and other sites may become affected. The causative virus (named therein camel PPV; CPPV) is a tentative member of the genus PPV that have not been approved as a species [11]. Nodules appear on the lips of affected animals followed in most cases by swelling of the face and sometimes the neck. Papules and vesicles appear later and within a few days develope into thick scabs and fissured crusts [4]. This disease results in high morbidity, but variable mortality rates. It has been reported in camels in the former USSR, Mongolia, Kenya, Somalia, Sudan, Arabian Peninsula and India [12, 13]. The disease was reported to cause a mortality rate reaching 9 % in the Sudan when it occurs in areas abundant in thorny acacia trees that cause abrasion to lips allowing replication of the virus [12]. In Iran, Mombeni [13] also reported 70 % morbidity and 6 % mortality rates aggravated by secondary bacterial infection and starvation due to mouth lesions. Contagious ecthyma was recently reported from Bahrain and Saudi Arabia [14], India [13] and Iran [15].

On the other hand, camel papillomatosis leads to a wart-like lesion often found around the lips and nostrils and may be misdiagnosed as a pox disease, especially when generalized lesions occur [16]. The disease appears as round, cauliflower-like horny masses mainly found on the skin of the lips and submandibular area and rarely on hind and forelimbs [17]. A recent publication [2] showed that the causative virus is unique and genetically different from the bovine papilloma virus.

The risk caused by these diseases is not only due to mortality, which can reach 28 % in camelpox [18] and 9 % in young camel calves in CCE [19], but also due to loss in milk and meat production because of calf loss, labor, and quality of skin. Besides, camelpox can cause human disease and the first conclusive evidence of zoonotic CMLV infection in humans, associated with outbreaks in dromedary camels has been recently reported in India [8].

As the lesions of CCE in affected animals are often indistinguishable from lesions caused by CMLV or CdPV, especially when these diseases co-exist in the same locality and when CCE undergoes a generalized course of disease, these diseases are not easy to differentiate from each other based only on clinical manifestations.

Camelpox is routinely diagnosed based on clinical signs, pathological findings and cellular and molecular assays. Five complementary techniques might be advised for camelpox diagnosis: transmission electron microscopy (TEM), cell culture isolation, standard polymerase chain reaction (PCR) assays, immunohistochemistry and demonstration of neutralizing antibodies. The laboratory diagnosis of these Pox and pox-like diseases of camels, with exception of camelpox remains an uneasy task due to difficulties to isolate and propagate the causative viruses of CCE and papillomatosis. Even for camelpox the current classical methods for laboratory diagnosis are unreliable and time-consuming (virus isolation in cell culture or embryonated chicken eggs) or not available (TEM) in countries where this disease is endemic.

Molecular methods such as PCR offer a better approach for the rapid diagnosis of Pox and pox-like diseases of camels and gel-based and real-time quantitative PCR (qPCR) assays have been developed. A PCR assay has been reported that can identify OPVs, including CMLV [20]. PCR assays have been developed for the detection of PPV infections [21, 22] and Khalafalla et al. [23] were the first to use PCR for diagnosis of CCE. For camel papillomatosis the pan papilloma virus specific primers have been used to detect virus genome and also used for genotyping [2].

The currently available PCR assays to identify CMLV are based on the detection of sequences encoding for the A-type inclusion body (ATI), the hemagglutinin (HA), the ankyrin repeat protein (C18L) or the DNA polymerase (DNA pol) genes [20, 24, 25]. ATI gene-based PCR has been performed with a single set of primer, which enables the differentiation of OPV species by producing amplicons of different sizes. Recently, a single-plex C18L and a duplex C18L-DNA pol PCR have been developed to specifically identify CMLV and to differentiate it from other OPVs, capripoxviruses (CaPV) and PPVs [26]. Recently, Venkatesan [27] reported the development of a qPCR for detection and quantitation of CMLV in clinical specimens.

Single target (monoplex) gel-based or real-time PCR assays established for Pox and pox-like diseases of camels require separate amplification of each virus genome. Therefore, there is a need to develop a single diagnostic test capable of precisely detecting multiple infectious agents simultaneously with comparable specificity and sensitivity instead of detecting each pathogen individually. In recent years, efforts have been made to combine monoplex assays into one multiplex format.

Correct diagnosis and differentiation of these diseases is essential for control programs. For instance, camelpox can be prevented using the available killed or live vaccines and infection by PPV could result in considerable losses particularly in the susceptible age group of less than one year [12]. In addition, camelpox can pose thread to public health due to its zoonotic nature [8].

In the present work, we report the development of a multiplex PCR method for simultaneous detection and differentiation of three important viruses of dromedary camels directly in DNA extracted from clinical specimens. We also evaluated the performance of the assay using clinical specimens collected from dromedary camels in Sudan and Saudi Arabia that showed pox-like lesions and demonstrated that the developed assay is specific, sensitive and would be attractive and practical method alternative to conventional protocols.

## Results

### Optimized parameters of the monoplex and multiplex PCR

**Fig. 2:**
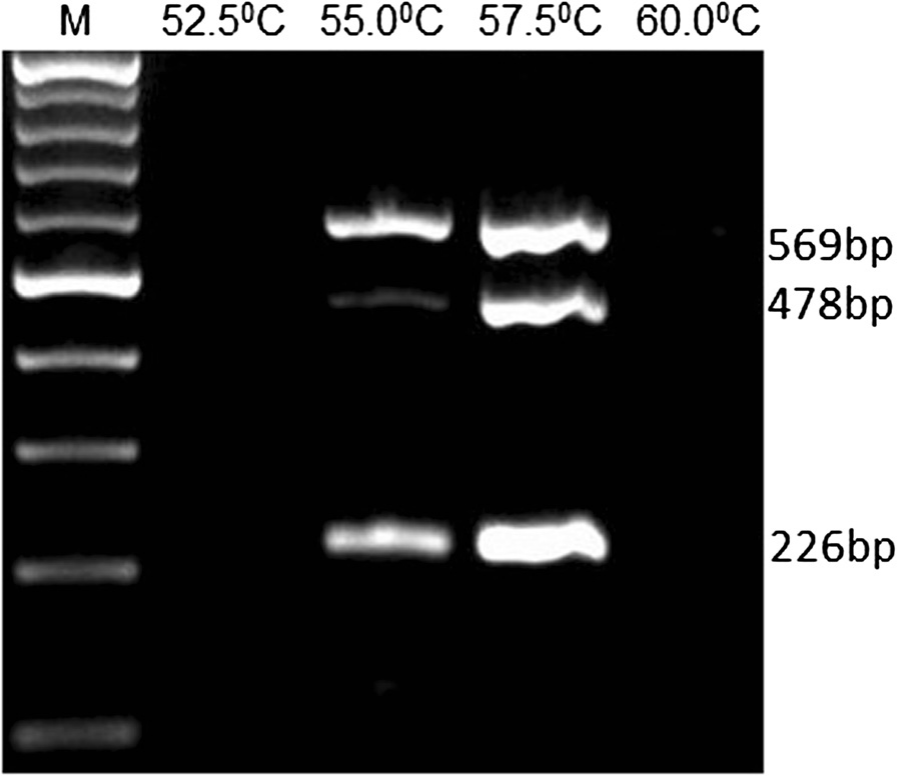
Agarose gel electrophoresis showing optimization of annealing temperature for the three viruses in the multiplex assay. A volume of 1 ul plasmid DNA containing 10^6^ plasmid copy numbers of CPPV, CdPV and CMLV was added to the PCR and the assay was run in a gradient thermal cycler (BIO-RAD 100) using different annealing temperatures indicated above the figure. PCR pruducts were 569, 478 and 226 bp for CPPV, CdPV and CMLV, respectively. Lane M; 100 bp marker. PCR products were resolved by electrophoresis in 1.5 % agarose in tris-acetate EDTA (TAE) buffer (40 mM Tris–acetate pH 8.0,1 mM EDTA) and the gel was stained with ethidium bromide and photographed CPPV; Camel parapox virus, CdPV; *Camelus dromedary* Papilloma Virus, CMLV; Camelpox virus

**Fig. 3:**
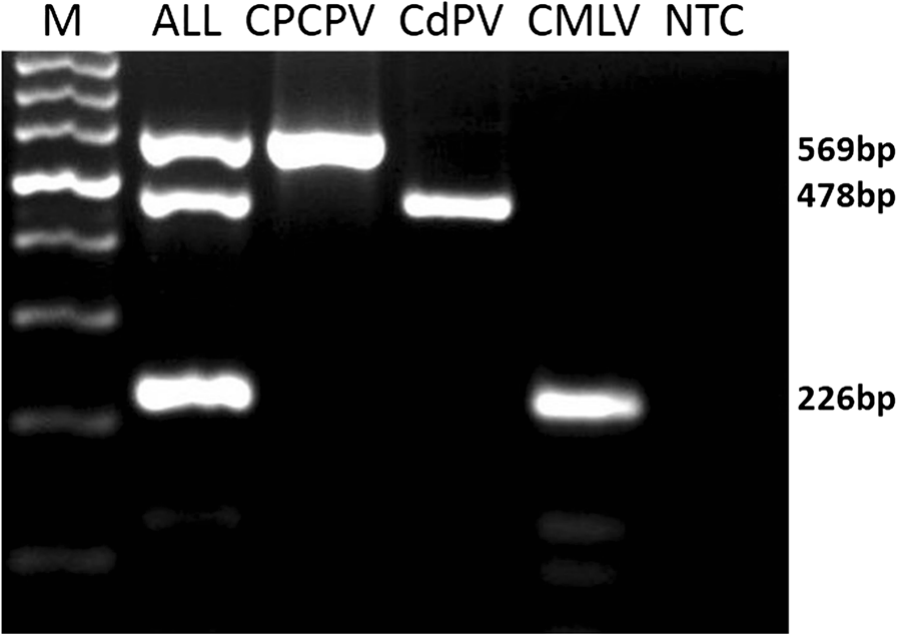
Agarose gel electrophoresis of the multiplex PCR amplified with plasmid DNA containing 10^6^ copy numbers of three viruses (CPPV, CdPV and CMLV). PCR was performed with the optimum reagent concentrations and conditions using multiple and single combination of the three plasmids.. Lane M; 100 bp marker, All; mixture of the three plasmid DNA. CPPV; Camel parapox virus, CdPV; *Camelus dromedary* Papilloma Virus, CMLV; Camelpox virus, NTC; non-template control (all PCR components except template DNA). PCR products were resolved by electrophoresis in 1.5 % agarose in tris-acetate EDTA (TAE) buffer (40 mM Tris–acetate pH 8.0,1 mM EDTA) and the gel was stained with ethidium bromide and photographed

The optimization process was performed using plasmid DNA construct containing the targeted gene fragments of the three viruses. The performance of the three sets of primers was first tested in an individual PCR assay before combining them in a multiplex format. The optimum annealing temperature of the multiplex PCR was found to be 57.5 °C (Fig. 2). The developed multiplex PCR gave visible DNA bands when a constant concentration of the three plasmid constructs (10^6^ copy number) containing insert of the targeted genes were tested separately and when all mixed together (Fig. 3). The assay also performed well using Qiagen Hotstart PCR kit (Qiagen, Germany) and Multiplex PCR Master Mix (New England Biolabs, UK). The thermal cycling was performed in a Tpersonal Thermal Cycler (Biometra, Germany).

### The specificity of the multiplex PCR

**Fig. 4:**
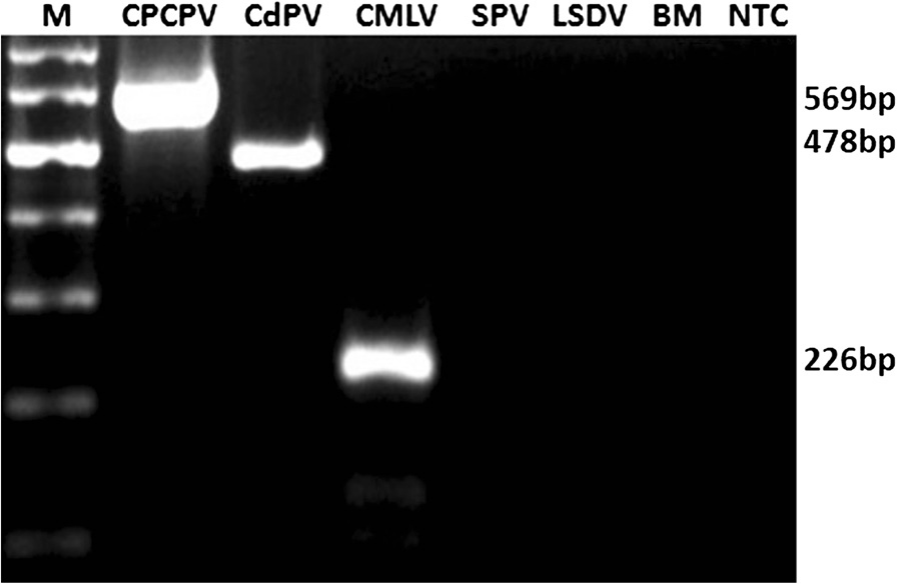
Specificity of the optimized multiplex assay in the detection of individual targets of CPPV (strain CCE41), CdPV (genotype CdPV1) and CMLV (strain CMLV-14) and unrelated DNAs extracted from SPV (*sheeppox virus*), LSDV (*Lumpy skin disease virus*) and BM (*Brucella melitensis*). PCR products were resolved by electrophoresis in 1.5 % agarose in tris-acetate EDTA (TAE) buffer (40 mM Tris–acetate pH 8.0,1 mM EDTA) and the gel was stained with ethidium bromide and photographed

The developed multiplex PCR was confirmed to be specific in amplifying DNA fragments of the three plasmid constructs and in detecting all the known CPPV, CMLV and CdPV DNAs described in Table 1, which were diagnosed initially by monoplex PCR. The assay produced amplicons of expected sizes that were distinguishable in the agarose gel; 569, 478 and 226 bp PCR products from DNAs of known CPPV, CdPV and CMLV, respectively while no positive signal was obtained when using *sheeppox virus* (SPV), *Lumpy skin disease viru*s (LSDV), *Brucella melitensis* (BM) or the no template control (NTC) (Fig. 4), suggesting that the assay was highly specific for detection of the target viruses. Furthermore, sequencing data confirmed the amplification of expected DNA sequences from the three viruses (data not shown).

**Table 1:** List of reference virus strains used in this study

SI No.	Virus ID	Virus ID/test	Collection place	Year	Reference
1	VD45	CMLV^1^	Niger	1981	Nguyen et al. (1989)
2	Ducapox	CMLV	UAE	1989	Kaaden et al. (1990)
3	CML1	CMLV	Iran	1970	Ramyar and Hessami (1972)
4	CMLV-14	CMLV	UAE	1994	Pfeffer et al. (1996)
5	CP/Mg/92/1	CMLV	Sudan	1992	Khalafalla et al. (1998)
6	CP/Nw/92/2	CMLV	Sudan	1992	Khalafalla et al. (1998)
7	CP/Db/92/3	CMLV	Sudan	1992	Khalafalla et al. (1998)
8	CP/Tm/93/6	CMLV	Sudan	1993	Khalafalla et al. (1998)
9	CMLV/115	CMLV	Sudan	1992	Abdellatif et al. (2013)
10	V1	CPPV^2^	Sudan	1991	Khalafalla et al. (1994)
11	CCE41	CPPV	Sudan	1993	Khalafalla et al. (2005)
12	CCE48	CPPV	Sudan	1993	Khalafalla et al. (2005)
13	CdPV1	CdPV^3^	Sudan	2009	Ure et al. (2011)
14	CdPV2	CdPV	Sudan	2009	Ure et al. (2011)

1 *CMLV*: camelpox virus, 2 *CPPV*: camel parapox virus, 3 *CdPV*: *camelus dromedary* papilloma virus

### The sensitivity of the multiplex PCR

**Fig. 5:**
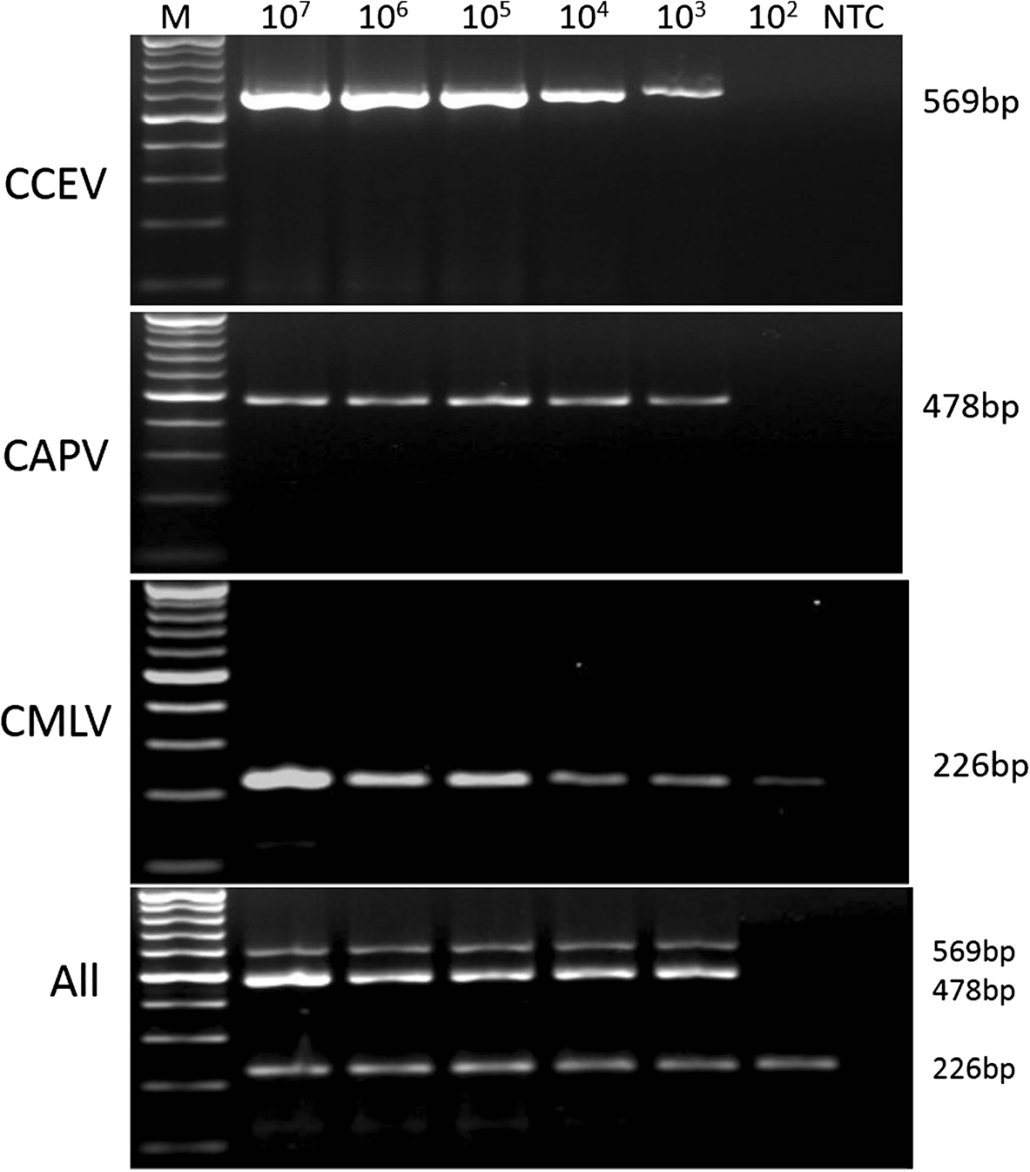
Agarose gel electrophoresis showing Limit of detection (LOD) of the monoplex and multiplex PCR assays employed over 10-fold serial dilutions of the three target gene-plasmids of CPPV, CdPV and CMLV. The concentrations of each plasmid DNA were indicated above each lane. PCR products were resolved by electrophoresis in 1.5 % agarose in tris-acetate EDTA (TAE) buffer (40 mM Tris–acetate pH 8.0,1 mM EDTA) and the gel was stained with ethidium bromide and photographed

The PCR sensitivity tests were performed using 10-fold serial dilution of the DNA plasmid constructs that were first diluted to contain 10^9^ copy numbers. DNA copy numbers ranging between 10 and 10^8^ were submitted to monoplex and multiplex PCR. The lower limit of detection (LOD) was found to be 10^3^ copy numbers of plasmid DNA for CPPV and CdPV and 10^2^ for CMLV (Fig. 5). On the other hand, the LOD was found to be 19 pg for DNA extracted from purified CMLV-14 reference strain (data not shown). The sensitivity of the monoplex and multiplex PCR assays was similar for CMLV, but the sensitivity of both assays in detecting CPPV and CdPV was 10 fold lower than for CMLV (Fig. 5).

### Analysis of clinical specimens with the multiplex PCR

**Fig. 6:**
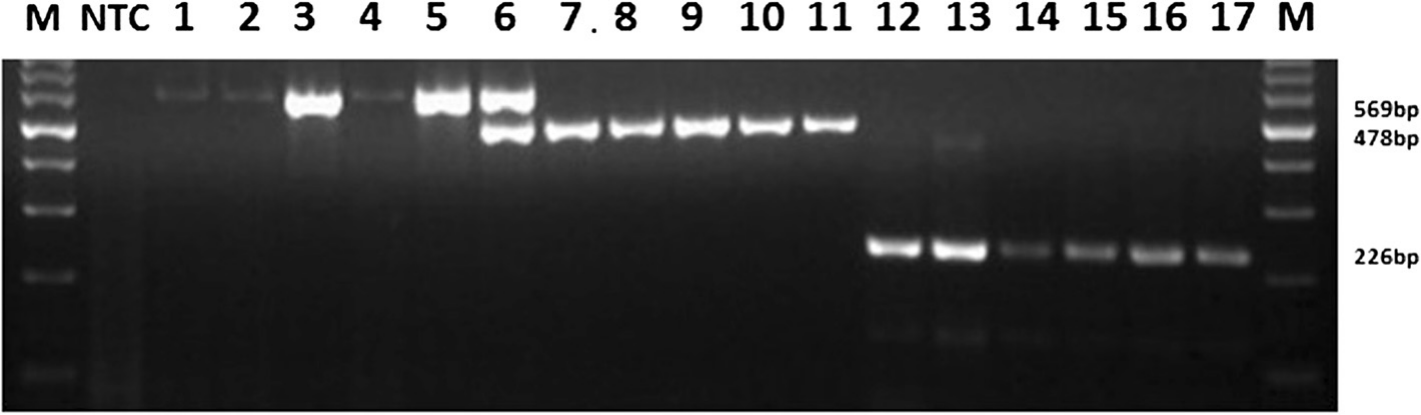
Agarose gel electrophoresis showing 17 positive field specimens detected by the developed multiplex PCR. A volume of 3 μl DNA was as template in a total reaction volume of 50 μl. Lane M; 100 bp marker, NTC; non-template control (all PCR components except template DNA), Lanes 1–5; specimens positive for CPPV, Lane 6; Field specimen with CPPV and CdPV co-infection, Lanes 7–11; specimens positive for CdPV, Lanes 12–17; specimens positive for CMLV. PCR products were resolved by electrophoresis in 1.5 % agarose in tris-acetate EDTA (TAE) buffer (40 mM Tris–acetate pH 8.0,1 mM EDTA) and the gel was stained with ethidium bromide and photographed

The multiplex PCR assay successfully amplified the expected DNA fragments from 86 clinical materials: CPPV (*n* = 42), CdPV (*n* = 27) and CMLV (*n* = 17) as shown in Table 2. Interestingly, the developed assay detected co-infection with CPPV and CdPV in one clinical specimen collected at *Showak* area of eastern Sudan in 2013 (Fig. 6). This result could only be detected by multiplex PCR as these specimens were previously diagnosed as CPPV positive by a monoplex PCR. The multiplex was able to detect CPPV, CdPV and CMLV DNAs in all analyzed clinical samples positive by monoplex PCR (Table 2).

**Table 2:** Testing clinical specimens to evaluate the performance of Monoplex and Multiplex PCR assays

Country/area/year	No. of skin specimens	Suspected virus	Monoplex PCR positive specimens (%)	Multiplex PCR positive specimens (%)	Positivity by standard methods
Sudan/Showak/2000	6	CMLV	6 (100)	6 (100)	ND*
Sudan/Showak/2013	8	CMLV	6 (75)	6 (75)	4 (50)
SaudiArabia/Hail/2014	3	CMLV	3 (100)	3 (100)	3 (100)
Sudan/Showak/1993	6	CPPV	4 (66.7)	4 (66.7)	3 (50)
Sudan/Showak/2000	8	CPPV	7 (87.5)	7 (87.5)	ND
Sudan/Showak/2005	6	CPPV	6 (100)	6 (100)	ND
Sudan/Showak/2012	6	CPPV	4 (66.7)	4 (66.7)	ND
Sudan/Showak/2013	5	CPPV	3 (60)	3 (60)	ND
Sudan/Showak/2013	8	CPPV	6 (75)	6 (75)	ND
Saudi Arabia/Hail/2014	3	CPPV	0	0	ND
Sudan/Showak/1994	5	CdPV	5 (100)	5 (100)	ND
Sudan/Khartoum/2009	6	CdPV	5 (83.3)	5 (83.3)	ND
Sudan/Showak/2012	4	CdPV	3 (75)	3 (75)	ND
Sudan/Showak/2013	3	CdPV	3 (100)	3 (100)	ND
Saudi Arabia/Al-Ahsa/2013	3	CdPV	0	0	ND
Saudi Arabia/Al-Ahsa/2014	6	CdPV	4 (66.7)	4 (66.7)	4
Total	86		65 (75.6)	65 (66.7)	

ND: not done

## Discussion

Search of the literature revealed that diagnosis of the published outbreaks of CCE was confirmed by TEM [16, 28–33], serology [34, 35] or gel-based PCR [13–15, 23]. For CdPV infection, diagnosis was achieved mainly by TEM [36,], histopathology [17, 36–38], and gel-based PCR [2]. On the other hand, various diagnostic techniques were employed for CMLV infection including virus isolation [7, 10, 39–52], TEM [48], immunohistochemistry [43, 49], gel-based PCR [3, 7–10, 23, 26] and real-time PCR [27]. Virus isolation in cell culture and embryonated eggs is time-consuming and besides, cannot be applied for diagnosis of CPPV and CdPV infections because these viruses are difficult to cultivate. Even if isolation is possible, this system may not identify multiple pathogens in a single clinical specimen because identification depends on the predominant causative agent and influenced by the selective cell type. EM is rapid and specific in distinguishing the three viruses, but the equipment needed is expensive and unaffordable to most laboratories in African and Asia where camels are raised.

In the short time since its inception, PCR has become an almost indispensable part of medical and diagnostic sciences. Due to extreme sensitivity, PCR methods have evolved to be the diagnostic method of choice [25, 53]. PCR assays to identify CPPV, CdPV and CMLV have been described, but to our knowledge, there is no report using this technique in a multiplex system. Virus-specific PCR assays that employs separate detection of each virus are costly compared to multiplex assays. Multiplex PCR is an approach commonly used to amplify more than one DNA target regions in a single PCR reaction.

In the present study, we tested several approaches to develop a multiplex PCR strategy that detects simultaneously CMLV, CdPV and CPPV. Previously published primers were tested in a monoplex and multiplex format and we selected the best performing published primer sets for CdPV and CPPV. Since most of the published OPV genus-specific primer sets produce relatively long PCR products that affect the sensitivity of the PCR [53] we designed primer sets that specifically detect CMLV HA gene with 226 bp product size. In our early PCR trials, optimum amplicon yield has not achieved possibly due to sub-optimal specific amplification that might occur because of the use of degenerate primer sets for CdPV. Degenerate primers are the only option available to detect Papilloma virus DNA because of the broad genetic heterogeneity [54]. Therefore, we used a hot-start (HS) DNA polymerase as this is one of the most important strategies to prevent such unwanted non-specific PCR products at low temperatures during PCR [55]. The developed multiplex PCR method was able to simultaneously detect CPPV, CdPV and CMLV DNAs. The assay was validated with viral genomic extracted from known virus isolates and DNA extracted from homogenized clinical skin specimens. The assay was able to correctly detect the target pathogens by amplification of targeted genes, even in case of co-infection.

In camels, the occurrence of mixed infections of camelpox and contagious ecthyma was previously reported [6] using electron microscopy. In the present study, we report the first co-infection of dromedary camels with CPPV and CdPV. This is of particular interest because mixed infection usually misses correct diagnosis owing to virus load in the most prominent lesion. Therefore, a rapid, cost effective and precise test that can simultaneously differentiate these viruses at an early stage is essential to prevent the spread of these diseases.

The developed multiplex PCR when used to test control positive plasmids and viruses as well as various clinical specimens, showed high specificity, sensitivity comparable to monoplex assays. The sensitivity of both assays in detecting CPPV and CdPV is 10 fold lower than for CMLV. This may be attributed to the fact that HA primers amplify relatively shorter fragments of PCR and hence resulted in a higher sensitivity [53].

To evaluate the diagnostic sensitivity results of the developed multiplex PCR were compared with standard techniques in a subset of the clinical specimens. Among 11 clinical specimens collected in 2013 and 2014 from dromedary camels in the Sudan and Saudi Arabia (Table 2), seven (63.6 %) were positive for CMLV by virus isolation in Vero cells culture, while nine (81.8 %) were positive in the developed multiplex PCR. Among six specimens collected in the Sudan in 1993 (Table 2) the transmission electron microscopy (TEM) technique previously detected PPV particles suggestive of CCE in three specimens (50 %) [23], while the developed assay detected four positive specimens (66.7 %) (Table 2). On the other hand, four specimens out of six (66.7 %) collected in Saudi Arabia in 2014 (Table 2) were found positive in both histopathology and the developed assay. These data show that the developed assay has a higher diagnostic performance and would be a reliable substitute for the detection of these viral diseases of camelids in clinical specimens.

## Conclusion

The development of a multiplex PCR method for the simultaneous detection of CMLV, CPPV and CdPV has been described and evaluated. The method is rapid, specific and sensitive and has a performance comparable to conventional monoplex PCR assays.

## Methods

### Viruses, DNAs and clinical specimens

Known viruses and DNAs used as positive controls for each pathogen in the present study are listed in Table 1. These include four reference CMLV strains: VD45 previously supplied by CIRAD-EMVT, France [56], Dubai camelpox vaccine (Ducapox), and DNAs extracted from purified CMLV-1 and CMLV-14 (kindly provided by Dr. Sophie Duraffour, Rega Institute, leuven, Belgium). Additionally, some previously published CMLVs isolated from outbreaks of the disease in Sudan [49], CPPV positive specimens collected from previous outbreaks [19] and skin specimens from an outbreak of papilloma infection in the Sudan [2], as well as the vaccine strain Sudan CMLV/115 [57] were also included.

*Sheeppox virus* (SPV) strain SGP0240, *Lumpy skin disease virus* (LSDV) strain isolated in the Sudan [58] and *Brucella Melitensis* (BM) strain REV 1 original seed (kindly provided by Dr. Iaam El Sanousi, Veterinary Research Institute, Sudan) were used as negative controls and in the specificity experiments.

To validate the specificity and sensitivity of the developed assay a total of eighty-six skin scabs and nodules which were collected from dromedary camels in Sudan and Saudi Arabia showing symptoms of pox and pox-like lesions between 1993 and 2014 (Table 2).

### Tissue homogenization and DNA extraction

A 20 % suspension was made of the scab material in tris-EDTA (TE) buffer (pH 7.4), freeze-thawed at −30 °C, mechanically homogenized using a mechanical homogenizer (TissueRuptor, Qiagen, Germany) and centrifuged at 1500 g for 10 min at 4 °C. Total viral DNA was extracted from 200 μl of each sample supernatant using GF-1 Viral Nucleic Acid Extraction Kit (Vivantis Technologies, Malaysia) according to manufacturer instructions. DNA was also similarly extracted from supernatant of Vero cells infected with CMLV. Bacterial DNA was extracted from *Brucella Melitensis* strain REV culture by a bacterial extraction kit (Vivantis Technologies, Malaysia).

### Oligonucleotide primers

The multiplex PCR assay included two sets of published primers previously described in the literature, but had never been used simultaneously. The primer set PPP-1/PPP-4 [21] amplifies a 569 bp region of the conserved *major viral glycoprotein* (*B2L*) gene of PPV, which is a widely used in PPV diagnosis and genotyping. For the *camelus dromedary* papilloma virus (CdPV), we used the degenerate pan-papilloma virus specific primers described by Forslund et al. [54] that target the L1 ORF of PV (Table 3). For the CMLV, primer sets targeting genes encoding for the *A-type inclusion body* (ATI), the *hemagglutinin* (HA), the *ankyrin repeat protein* (C18L) and the DNA polymerase were initially tested in combination with the above-mentioned primer sets, but results were not satisfactory. Therefore, new pair of primers targeting the *hemagglutinin* (HA) of OPV was designed. Sequences of CMLV HA gene originating from different countries were obtained from the Genbank®. Regions of high homology in different CMLV strains were identified by sequence alignment using Clustal-O (http://www.ebi.ac.uk/Tools/msa/clustalo/). Primers were initially selected by using Primer Explorer V4 (http://primerexplorer.jp/e/) and then manually edited. Details on primer sets used in the present study are shown in Table 3.

**Table 3:** Details of oligonucleotide primers used in this study for Multiplex PCR and for construction of standard plasmids

SI No.	Name	Sequence (5′-3′)	Target virus	length	PCR product size	Reference
1	PPP-1	GTCGTCCACGATGAGGAGCT	CPPV	20	594 bp	Inoshima et al. (2000)
2	PPP-4	TACGTGGGAAGCGCCTCGCT		20		
3	FAP59	TAACWGTNGGNCAYCCWTATT	CdPV	21	478 bp	Forslund et al. (1999)
4	FAP64	CCWATATCWVHCATNTCNCCATC		23		
5	HA F3	ACAGTAAGTACATCATCTGGA	CMLV	21	226 bp	This study
6	HA R3	TCGTGATGTTTTCTACAGTTG		21		

### The monoplex PCR

PCR amplifications of the B2L gene of PPV and L1 ORF of PV were performed with primers shown in Table 1 in a total reaction volume of 25 μl as described by Inoshima et al. [21] and Forslund et al. [54], respectively. For the amplification of the CMLV HA gene PCR reaction was performed in a final volume of 25 μl that contained 1× PCR buffer (Vivantis Technologies, Malaysia), 10 mM dNTPs mix, 0.4 μM of each primer (HA-F and HA-R), one unit Taq DNA polymerase (Vivantis Technologies, Malaysia) and one μl DNA template. The PCR amplification was carried out in a Tpersonal Thermocycler (BIOMETRA, Germany) under the following conditions: initial denaturation at 94 °C for three min followed by 35 cycles each included denaturation step at 94 °C for 30 s, annealing step at 56 °C for 30 s and extension step at 72 °C for 30 s. A final extension step at 72 °C for seven min was included. PCR products were resolved by electrophoresis in 1.5 % agarose in TAE buffer (40 mM Tris–acetate pH 8.0,1 mM EDTA) and the gel, stained with ethidium bromide and photographed using ultraviolet gel documentation system (BIOMETRA, Germany).

### Plasmid template construction

As quantitative calibration standards, three plasmids containing targeted DNA fragments were prepared. PCR assays were carried out to amplify selected genes using monoplex protocols described above from reference strains CCE 41 (CPPV), CdPV1 (CdPV) and CP/Mg/92/1 (CMLV) (Table 1). The PCR products were separated by agarose gel electrophoresis, purified with a PCR purification kit (Vivantis Technologies, Malaysia) and the PCR product was cloned into pBASE/TA vector. The ligation product was transformed into JM109 E.coli strain. Positive clones were identified and the plasmid purified using a commercial kit and sent for sequencing in an automated ABI 3730 DNA sequencer (Applied Biosystems, USA) using the BigDye® Terminator v3.1 cycle sequencing kit chemistry.

### Optimization of the multiplex PCR

A number of experiments were carried, several chemical and thermal conditions were evaluated, and the assay optimized by adjusting primers and MgCl_2_ concentrations as well as the thermal cycling temperatures and duration. The best conditions were established based on amplicon yield and specificity. Monoplex PCR experiments were carried out before combining them in multiplex assays. Ordinary and hot-start Taq DNA polymerases were tested. PCR was tested with conventional PCR approach in addition to a hot-start amplification that employed heating all PCR components excluding the Taq DNA polymerase at 94 °C and then addition of the enzyme. The multiplex PCR was performed in 50 μl volume containing 1 μL (10^6^ copy number) of plasmid DNA, 0.8 μM of PPV primers (PPV-1 and PPV-4) and papilloma virus (PV) (FAP 59 and FAP64), 0.4 μM of OPV primers (HA-F and HA-R), 1× PCR buffer (Vivantis Technologies, Malaysia) including 160 mM (NH_4_)_2_ SO_4_, 500 mM Tris–HCl (pH 9.2),17.5 mM MgCl_2_ and 0.1 % Triton™ ×-100) and 10 mM dNTPs mix. A manual hot-start procedure was followed in which the above PCR components were heated up to 95 °C for two min and one unit of Chrome Max Taq DNA polymerase (Vivantis Technologies, Malaysia) was added at the same temperature followed by incubation at 95 °C for nine min, 30 cycles of denaturation (94 °C, one min), annealing (57.5 °C, one min) and extension (72 °C, one min) and a final extension at 72 °C for 10 min. The cycling program for the multiplex PCR has eight steps with a total running time of two h, 34 min and 18 s. PCR products were resolved by electrophoresis in 1.5 % agarose in tris-acetate EDTA (TAE) buffer (40 mM Tris–acetate pH 8.0,1 mM EDTA) and the gel was stained with ethidium bromide and photographed. For gel detection, eight μl of PCR product was stained with ethidium bromide and resolved on a 1.5 % agarose gel using TAE buffer.

### Specificity and sensitivity

To estimate the specificity, the developed multiplex assay was tested with all the three plasmid constructs (10^6^ copy number), known CPPV (8 ng), CdPV (20 ng) and CMLV (10 ng), SPV (20 ng), LSDV (20 ng) and BM (15 ng) DNAs in 1 μl templates volume. Furthermore, the expected PCR products obtained from the experiments were sequenced to evaluate the specificity of the assay.

The sensitivity of multiplex PCR and the corresponding monoplex PCR was performed using serial 10-fold dilution of the plasmid constructs. Plasmid constructs were first reconstituted in molecular grade-H_2_O, quantified (NanoDrop-1000, Thermo Fisher Waltham, USA), 10-fold serially diluted to contain concentrations of 10-10^8^ copy numbers /μl and then submitted to monoplex and multiplex PCR. Furthermore, 10-fold serial dilution was carried out for DNA extracted from purified cell culture-strain of CMLV (CML-14) to evaluate the sensitivity of the monoplex and multiplex PCR.

### Applicability of the multiplex PCR

To validate the developed multiplex PCR assay successfully amplified the expected DNA fragments from 86 DNA samples extracted from homogenized clinical skin specimens as shown in Table 2. A volume of 3 μl DNA was as template in a total reaction volume of 50 μl. All collected CCE, camelpox and camel papillomatosis clinical samples were previously tested using PPV-specific PCR [21], OPV-specific PCR [59] and Pan-Papilloma PCR [54] and were confirmed as CPPV, CdPV and CMLV infections, respectively.
